# Gas Chromatography-Atmospheric Pressure Chemical Ionization (GC-APCI) Expands the Analytical Window for Detection of Large PAHs (≥24 Ringed-Carbons) in Pyroplastics and Other Environmental Matrices

**DOI:** 10.1021/acsomega.5c11703

**Published:** 2026-02-08

**Authors:** Cara Megill, Douglas M. Stevens, Christopher M. Reddy, Bryan D. James, Robert K. Nelson, Frank L. Dorman

**Affiliations:** † Department of Chemical Engineering, 1848 Northeastern University, Boston, Massachusetts 02115, United States; ‡ 36565 Waters Corporation, 34 Maple Street, Milford, Massachusetts 01757, United States; § Department of Marine Chemistry and Geochemistry, 10627 Woods Hole Oceanographic Institution, Woods Hole, Massachusetts 02543, United States; ∥ Department of Chemistry, 3728 Dartmouth College, Hanover, New Hampshire 03755, United States

## Abstract

Open waste burning, large-scale fires, and maritime disasters produce partially burnt plastic called “pyroplastic”. Chemical markers would provide a complementary method to appearance and physical properties for identifying pyroplastics in environmental samples, particularly with respect to microplastics. Pyroplastic can contain significant quantities and unique distributions of parent polycyclic aromatic hydrocarbons (PAHs) with molecular weights up to 278 Da. Because of this enrichment, we considered whether large PAHs (≥24 ringed-carbons) could serve as chemical markers for pyroplastics. To address this, we developed a high-temperature method for gas chromatography atmospheric pressure chemical ionization (GC-APCI) coupled with tandem mass spectrometry (MS/MS) to target large PAHs with molecular weights ranging from 314–424 Da. Method development was performed using National Institute of Standards and Technology standard reference materials (SRMs) previously characterized for PAHs greater than 302 Da. A PAH class-specific MS/MS acquisition scheme combined with a simple, generic microextraction provided sensitive and specific detection without the need for sample fractionation or cleanup. Pyroplastics collected during the 2021 M/V *X-Press Pearl* ship fire and plastic spill were analyzed. A semiquantitative comparison showed that the pyroplastic samples contained over 2 orders of magnitude more of the 16 large PAHs (314–424 Da) than unburnt plastic pellets, reflecting previously observed trends for parent PAHs up to 278 Da. Qualitative comparison of samples and SRMs revealed multiple potential candidates (including 1,3,5-triphenylbenzene) suitable for further study as markers of pyroplastics in complex environmental samples. A suite of chemical markers for pyroplastics should prove helpful in monitoring efforts for air quality, waste management, microplastic pollution, and fires at the forest–urban interface.

## Graphical abstract

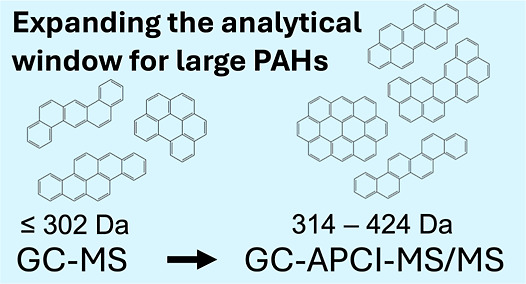

## Introduction

The measurement and reporting of polycyclic aromatic hydrocarbons (PAHs) are routine practices in environmental monitoring and risk assessment.^1–3^ However, of the thousands of PAHs, only the 16 designated as Priority Pollutants by the U.S. Environmental Protection Agency (EPA16) are typically reported.^4,5^ These span two to six ringed parent PAHs, ranging in molecular weight from 128 to 278 Da. It is well recognized that this limited analyte list drastically simplifies and misses a significant fraction of PAHs.^4^ For example, the concentration of C_1_–C_4_ alkylated PAHs can be greater than or equal to the concentration of the EPA16 in crude oils and fuels.^6^ Expanding the analytical window of PAHs has proven valuable for forensic analysis of oil spills (e.g., alkylated and heterocycle-containing PAHs) and relevant for toxicological evaluation­(e.g., oxygen-, nitrogen-, and sulfur-containing PAHs).^3,7^ Moreover, the sources of PAHs have distinct compositions of PAHs,^1,8^ which enables the use of PAHs for source apportionment.^2,7^ Including alkylated and sulfur-containing PAHs (those beyond the EPA16) in the investigation of environmental samples and other matrices provides greater discriminating power between potential sources (using extracted ion chromatograms).^3,7,9^ Additionally, monitoring the compositional changes of PAHs in the environment can inform about the fate and environmental processing of the matrix (e.g., oil weathering).^2^ While these efforts have made strides in considering other PAHs, an environmentally relevant fraction of PAHs remains underutilized.

PAHs with more than six rings have been reported in diverse environmental matrices.^10,11^ These large PAHs (≥24 ringed-carbons^10^) have been found in combustion-derived particulate matter,^12–19^ urban dust,^20–23^ refinery deposits,^11^ coal tars and pitches,^24–27^ cokes,^28^ soils,^20,22^ sediments,^20–23^ and deep-sea hydrothermal vent bitumen.^29^ Most reports of large PAHs in environmental matrices have been from analyses of U.S. National Institute of Standards and Technology (NIST) standard reference materials (SRMs);^18,21–23,25,26,30^ a few 302 Da isomers have been certified in three SRM.^31^ Nonetheless, isomers ranging from 326 to 374 Da have been reported in SRMs for urban dust (1649a/b), diesel particulate (1650b and 2975), coal tar (1597a), and marine sediment (1941b), displaying qualitative differences between the sources in their large PAH compositions;^18,20,22,23,27^ thus, large PAHs should offer forensic utility.

The detection of large PAHs in urban dust and marine sediments indicates the potential for our exposure to these compounds. Generally, the larger the PAH, the greater its toxicity.^32^ Limited investigations suggest that large PAHs are of toxicological significance, finding that an appreciable portion of the total toxicity of PAH-containing environmental matrices can be attributed to them,^13,33–35^ and that their toxicity can be potent.^32,36,37^ Despite their known occurrence and potential toxicity, the measurement of large PAHs is far from routine.^4^ Challenging their broader study has been the low levels of large PAHs in environmental samples, a considerable number of isomers, inaccessibility by conventional analytical methodologies and instruments (e.g., the EPA standard method for PAH analysis), and a lack of widely available analytical standards.^4^ Given their potential for toxicological harm, accessible analytical methods are needed for characterizing this overlooked fraction of PAHs in environmental matrices.

Due to their production during the incomplete combustion of organic matter, numerous possible global sources can produce large PAHs. In particular, the open burning of plastic poses a significant concern; it is estimated that more than 40% of municipal waste is burned in this manner.^38^ Recent work has shown that burning plastic creates a distribution of PAHs distinct from those in petroleum and from burning biomass.^39^ Additionally, triphenylbenzenes, specifically the 1,3,5-isomer, have been used as atmospheric tracers for the burning of polyethylene plastic and are recommended for inclusion in PAH analysis and monitoring strategies to track emission sources of burning plastic.^40–42^ To date, the most extensively studied burnt plastic, also known as “pyroplastic”, has been the material released into the ocean off the coast of Sri Lanka during the 2021 M/V *X-Press Pearl* ship fire and subsequent plastic spill.^39,43–46^ This event was the largest plastic spill in history,^47^ releasing upward of 1600 tons of plastic pellets and pyroplastic debris (Figure 1). Solvent extracts of the pyroplastics have been analyzed by one-dimensional gas chromatography mass spectrometry (GC/MS) for PAHs (parent homologues ≤278 Da),^39^ comprehensive two-dimensional gas chromatography high resolution time-of-flight mass spectrometry (GC × GC-HRT) for chemical complexity,^43^ and toxicological potential.^46^ These analyses revealed that the pyroplastic pieces had pyrogenic and petrogenic PAH signatures^39^ and had the greatest PAH content of any marine debris reported (∼200,000 ng/g),^39^ far exceeding established risk assessment thresholds for toxicological concern.^39^ The pyroplastic is expected to contain large PAHs and potentially have a unique composition.

**Figure 1:**
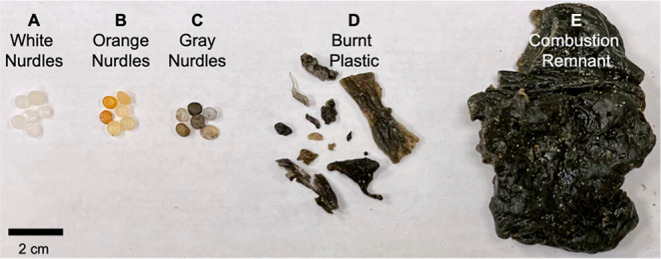
A photograph of representative white nurdles (A), orange nurdles (B), gray nurdles (C), pieces of burnt plastic (D), and larger combustion remnant chunks (E) collected from Pamunugama Beach, Sri Lanka, following the M/V *X-Press Pearl* ship fire and plastic spill. Orange and gray nurdles were not included in the present study. Reprinted from James et al.^39^ (CC BY-NC-ND 4.0).

Recent advances in soft ionization within an atmospheric pressure chemical ionization source for gas chromatography–mass spectrometry (GC-APCI) are well-suited for the analysis of large PAHs. Two contributing factors for this are the tolerance for high carrier gas flow rates by GC-APCI and the sensitivity of charge exchange APCI for aromatic species.^48^ Increasing the carrier gas flow rate reduces the elution temperature of analytes, making many higher mass analytes accessible using a conventional GC and column, although poor thermal stability may still pose challenges for some analytes.^49^ Classic vacuum-source GC/MS systems using electron ionization (EI) restrict the upper carrier gas flow allowed from the GC, and therefore, cannot fully utilize high GC carrier gas flows. The sensitivity of GC-APCI for components of petroleum, such as PAHs, has been previously reported.^48^ Furthermore, unlike LC-APCI, which commonly exhibits protonation of analytes in positive ion mode,^50^ GC-APCI+ can operate with dry source conditions. These conditions result in nitrogen-mediated charge exchange ionization and ions of the form M+^**•**^, the same form of molecular ion created using EI. As a result, because the charge site location on an ion dictates which product ions will be stable, the same multiple reaction monitoring (MRM) transitions can be used for both GC-APCI MS/MS and EI MS/MS.^51^

Herein, a GC-APCI method was developed for the detection of large PAHs. Due to the limited availability of pure standards, previously characterized NIST SRMs were used in method development as sources of large PAHs. The values of the method and detection of large PAHs were demonstrated by analyzing environmental plastic samples collected after the M/V *X-Press Pearl* ship fire and plastic spill.

## Materials and Methods

### SRMs and Environmental Samples

Several NIST SRMs were used for method development, including SRM 1597a Complex Mixture of Polycyclic Aromatic Hydrocarbons from Coal Tar (liquid), SRM 1991 Mixed Coal Tar/Petroleum Extract (liquid), and SRM 1649a Urban Dust (solid). Solid 1,3,5-triphenylbenzene (1,3,5-TPB; Fisher Scientific, purity >99%). The plastics investigated for large PAHs were subsamples of material previously reported on by members of our team.^39,43,45,46^ The plastic samples were collected on May 25, 2021, from Pamunugama Beach, Sri Lanka, following the M/V *X-Press Pearl* ship fire and plastic spill. The subsampled plastic included white pellets (also known as “nurdles”) and two forms of pyroplastic: burnt plastic and combustion remnants.

### Sample Preparation

SRM 1649a and the plastic samples were extracted using previously published methods^39^ for parity, with the exception that a single solution of deuterated PAHs was used as a surrogate. SV Internal Standard Mix (Restek) at 4000 μg/mL containing 1,4-dichlorobenzene-*d*_4_, naphthalene-*d*_8_, acenaphthene-*d*_10_, phenanthrene-*d*_10_, chrysene-*d*_12_, perylene-*d*_12_ was diluted to 200 ppb in methylene chloride (DCM; Acros Organics, 326760010; purity >99.9%) for use as the extraction solvent. SRMs 1597a and 1991 were diluted 10:1 in the extraction solvent, and the mass of 100 μL was recorded. A 1 mg/mL solution of 1,3,5-TPB was prepared in toluene (Sigma-Aldrich, 32249, purity ≥99.7%). A calibration curve of 1,3,5-TPB was created to quantify the compound in NIST SRM 1597a and environmental plastics (Figure S1).

### GC-APCI

The solvent extracts and dilutions were analyzed by GC-APCI on a Xevo TQ Absolute (Waters Corporation) tandem quadrupole mass spectrometer (TQ-MS/MS). The atmospheric pressure gas chromatography (APGC) ionization source was operated in charge transfer mode using dry N_2_ as the reagent gas. Dry N_2_ reagent gas creates molecular ions of the form M+^**•**^ with no significant adduct formation for large PAHs. The high reagent gas flow rate of 350 mL/min into the ionization chamber, combined with low energies applied to the transfer optics from the atmospheric pressure region to the first quadrupole, leads to a high molecular ion survival rate. Furthermore, the use of MS/MS with quadrupoles operating at unit mass resolution prevents potential effects on response caused by adduct formation. Acquisitions were performed in positive ion, MRM mode. Two MRM transitions were used for each of the 16 precursor masses, ranging from 314 to 424 Da (Table S1). No optimization of source conditions specific to the analysis of large PAHs was required. The list of 16 specific precursor masses targeted in this range was developed using multiple sources reporting large PAHs in NIST SRMs and samples from deep-sea hydrothermal vents.^18,29,30^ The two transitions represented constant neutral losses of 2 and 4 Da at high collision energies of 60–100 eV, using N_2_ as the collision gas.

For 1,3,5-TPB analysis, the APGC ionization source was operated in charge transfer mode using N_2_ as the collision gas. Acquisitions were performed in positive ion, MRM mode, yielding six MRM transitions for analysis (Table S2). Similar to the large PAHs, this analyte also shows neutral losses of 2 and 4 Da, reflecting behavior like that of the more condensed structures.

The 8890 GC (Agilent Technologies) was configured with N_2_ as the carrier gas and an Rxi-5HT column (Restek) of 15 m in length, 0.25 mm inner diameter, and 0.10 μm film thickness. The split/splitless (SSL) injection port was operated at a 10:1 split ratio with a temperature of 380 °C. A 1 μL injection volume was used for all analyses. The temperature program was 30.8 min; it started by holding at 40 °C for 0.5 min, then ramped to 160 °C at 14 °C/min, followed by a ramp to 395 °C at 22 °C/min and a hold at the temperature for 11 min. The flow rate of the N_2_ carrier gas was initially 0.60 mL/min, then ramped at 0.015 mL/min^2^ to 0.90 mL/min, followed by a ramp at 0.150 mL/min^2^ to 3.0 mL/min. To adapt the system for high-temperature work, the SSL was configured with a high-temperature septum (400 °C maximum), a 100% graphite liner O-ring, and a straight 4 mm inner diameter, wool-packed liner (450 °C maximum). Additional instrument and method details are included in the Supporting Information.

## Results

### Method Development for Analysis of Large PAHs by GC-APCI

A challenge to studying large PAHs has been a lack of widely available analytical standards for many of them.^4,11^ To overcome this barrier, three NIST SRMs were used: 1597a, 1991, and 1649a, representing a complex mixture of PAHs from coal tar, a mixed coal tar/petroleum extract, and urban dust, respectively. These SRMs are explicitly intended for evaluating analytical methods for the determination of PAHs.^31^ For example, SRM 1649a is considered one of the most evaluated environmental matrices with respect to PAHs.^52^ Additionally, these SRMs represent environmental matrices of varying PAH origin and formation temperature.^1,8^ To optimize the cone voltage and collision energy values for each set of MRM transitions, repeat injections of these SRMs at different steps in voltage were made. The optimum voltages were chosen as the values that gave the highest response for the narrowest chromatographic peak in the extracted ion chromatograph for each precursor mass.

The presence of PAHs with molecular weights of up to 352 Da has been reported in SRM 1597a.^30^ Therefore, it was used to assess the initial method performance and direct method development for PAHs in the range of 314 to 424 Da. The latest eluting peaks, identified in 1597a using the final method, were observed between 20 and 22 min and appeared at masses of 398, 400, and 424 Da. Peaks were symmetric with widths of less than 5 s, indicating satisfactory chromatographic separation. The final GC oven temperature of 395 °C was maintained for 11 min at a flow rate of 3.0 mL/min of N_2_ to ensure the complete elution of high-boiling components that might be present in sample extracts and additional SRMs. Fifty-one lower molecular weight PAHs, ranging from 128 to 302 Da and covering over 3 orders of magnitude dynamic range from 1 to 300 ppb, were also acquired in the final method.^53^ This method resulted in the relatively rapid (within 30 min of injection), sensitive, and specific detection of large PAHs using N_2_ carrier gas (compared to relatively expensive and scarce He) from facile microextractions of environmental samples (without additional fractionation or cleanup).

Among large PAHs, the 302 Da isomers lay at the beginning of the set, having reported reference and certified values for several of them.^31^ Additionally, their extracted ion chromatograms have been compared between several NIST SRMs.^21^ Investigation of the 302 Da isomers revealed ∼13 peaks in the SRMs, with potential coelution for a few isomers (Figure S2). The SRMs 1597a and 1649a showed minor relative differences in their distributions of 302 Da isomers (Figure S2), in agreement with previous reports, which quantified 23 isomers.^21^ SRM 1991 was noticeably different compared to the other two SRMs, missing a discernible peak at ∼16.75 min for dibenzo­[*a*,*h*]­pyrene.

The three SRMs contained large PAHs with varying distributions, characterized by molecular weights of 326, 350, and 374 Da (Figure 2). Although complete chromatographic resolution is not achieved for all isomers, multiple peaks of different intensities hold potential for characterizing sample types, such as the peak at 17.74 min of 326 Da, the peaks at 18.24 and 18.52 min of 350 Da, and the peaks at 19.28 and 19.53 of 374 Da (Figure S3). Differences between the SRMs were to be expected, given their origins and formation temperatures.^1,8^

**Figure 2:**
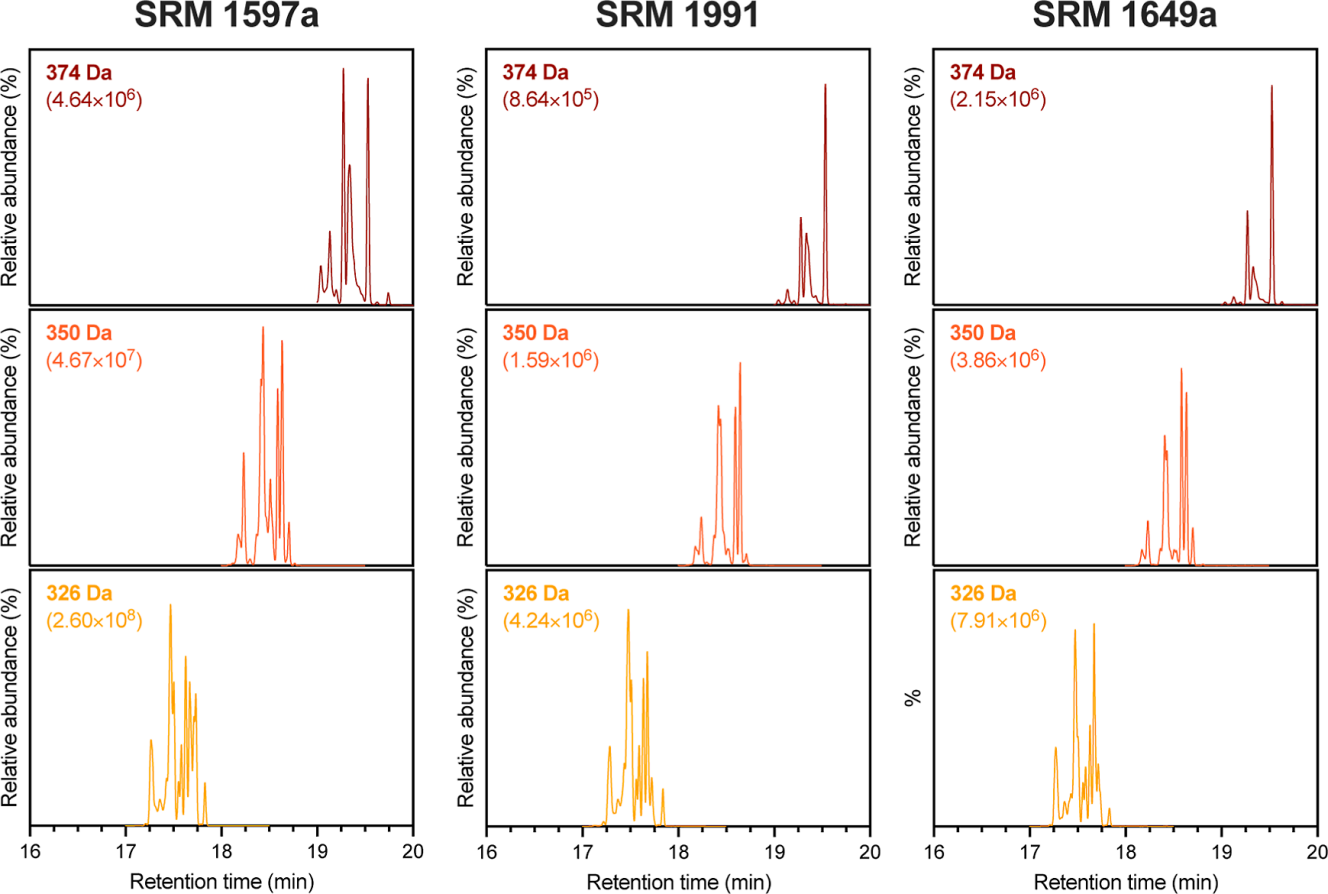
Comparison of the resolved isomers for 374, 350, and 326 Da of the three SRMs 1597a (coal tar), 1991 (mixed coal tar/petroleum), and 1649a (urban dust).

The GC-APCI method was also amenable to the analysis of 1,3,5-TPB. Multiple product ions were produced, adding specificity for the detection of this compound in environmental samples. Some studies have reported detecting 1,3,5-TPB in petrogenic sources (e.g., coal tar pitch).^54,55^ To assess the potential of a false-positive signal due to components of SRM 1597a coeluting with or mimicking 1,3,5-TPB, a pseudo-MRM trace at 5 eV was used to monitor all 306 Da analytes. No peaks were observed, indicating no presence of 1,3,5-TPB or confounders in SRM 1597a using this method. However, significant peaks were observed on this low specificity trace outside the acceptable retention range for this method. Alternative methods using different sample preparation, separation, and detection may result in these peaks being misassigned as 1,3,5-TPB. Furthermore, for the six additional MRM transitions monitored, none contained a chromatographic peak within a reasonable retention window (±0.10 min) of the 1,3,5-TPB retention time in the solvent standard and spiked SRM 1597a aliquot. This finding results in a nondetect of 1,3,5-TPB for an in-aliquot concentration down to the method limit of 0.2 ppb.

### Analysis of Large PAHs in Pyroplastic Samples by GC-APCI

Macroplastic and microplastic debris from the M/V *X-Press Pearl* ship fire and plastic spill were investigated for the presence and distribution of large PAHs. The field samples included white, unburnt polyethylene nurdles and two types of pyroplastic, termed burnt plastic and combustion remnant (Figure 1), both of which were polyethylene.^43^ The types of pyroplastics were previously operationally defined based on the size and shape of the pieces.^43^

Investigation of the 302 Da isomers revealed profiles similar to those of the SRMs; however, unique differences were observed (Figure S2). Eighteen to twenty-one peaks were detected across the plastic samples. Compared with the SRMs, the pyroplastics showed decreases in the relative abundance of dibenzo­[*a*,*e*]­fluoranthene and increases in dibenzo­[*a*,*h*]­pyrene (Figure S2). This trend was not the case for the white nurdle sample. Starting at a retention time of 16.80 min, two peaks were unique to the pyroplastic samples (not present in the SRMs and especially minor in the white nurdles). These peaks followed that for dibenzo­[*a*,*h*]­pyrene, which had previously been reported as the last detected 302 Da isomer compound in the SRMs,^21^ supporting their novelty as marker compounds of pyroplastics.

The plastic samples displayed similarities and differences in the presence and distribution of large PAHs (Figure 3). The white nurdles, burnt plastic, and combustion remnant all contained high-intensity peaks at 20.50 min for 398 Da and 19.55 min for 374 Da. Peaks between 20.00 and 20.25 min of 398 Da and the 19.35 min peak of 374 Da were reduced in intensity for the pyroplastics compared to the white nurdles (Figure S3). Conversely, the peaks between 18.50 and 18.75 min of 350 Da were more intense in the pyroplastics compared to the white nurdles.

**Figure 3:**
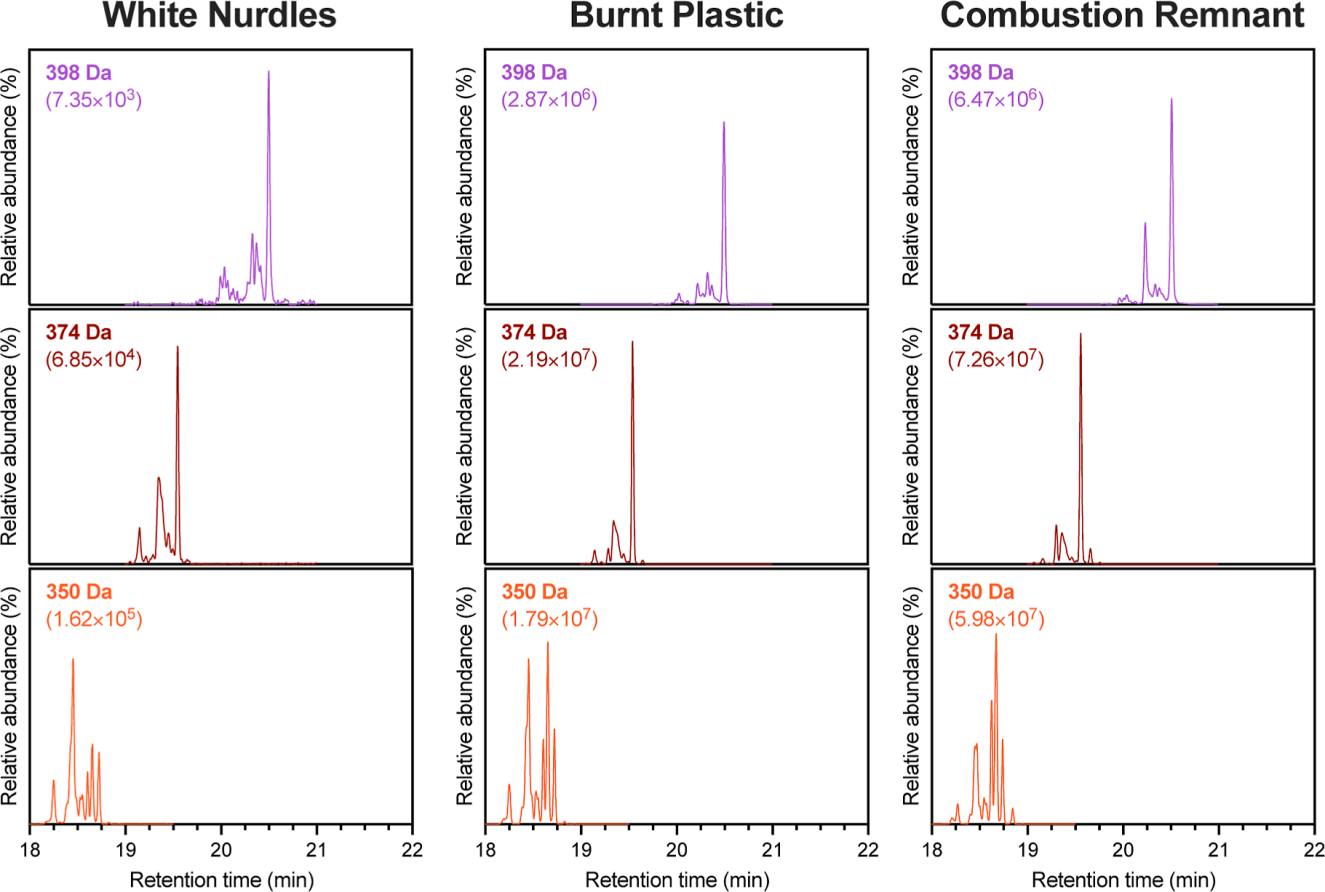
Comparison of the resolved isomers for 398, 374, and 350 Da of the white nurdles, burnt plastic, and combustion remnant pieces collected from Pamunugama Beach, Sri Lanka, following the 2021 M/V *X-Press Pearl* ship fire and plastic spill.

The levels of all the targeted large PAHs were at least 2 orders of magnitude higher in the pyroplastics than in the white nurdles. These levels included the individual PAHs that were common to the pyroplastics. This trend mirrored that observed for smaller PAHs and the solvent-extractable content of the sample types.^39,43,46^ There were also slight differences in the signatures of the large PAHs found in the pyroplastics. The 398 Da peak at 20.24 min, along with the 374 Da peak at 19.31 min and the 350 Da peak at 18.61 min, were more prominent in the combustion remnant compared to the burnt plastic (Figure S3).

Similar to the differences in large PAH signatures between the SRMs, the plastic samples had different signatures. Such qualitative interpretation of extracted ion chromatograms is forensically accepted for distinguishing sources.^3^ This feature can potentially be utilized to differentiate between plastic types (e.g., virgin, weathered, and partially combusted) as well as to identify sources of the large PAHs and the combustion temperature.

Comparing the distributions of isomeric classes revealed similarities and differences between the plastic samples and the SRM 1597a, as expected (Figure 4). In all samples and in SRM 1597a, the 326 Da isomers had the highest relative abundance. When comparing the abundance of the large PAHs greater than 348 Da, the relative amounts of 350 and 352 Da isomers flipped between the SRM 1597a and plastics. Thus, the ratio of these isomers may hold diagnostic utility for source apportionment. Additionally, we observed an abundance of the 374 and 424 Da isomers in the plastic samples, whereas they were much less abundant in SRM 1597a. Notably, we propose the 424 Da isomers as a potential marker of burnt plastics as the relative abundance is much greater in the burnt nurdles and combustion remnants than in the white (unburnt nurdles) and the 424 Da isomers are proportionally nearly absent in the SRM 1597a, indicating this isomer has the potential to be used diagnostically for identifying pyrogenic sources (e.g., the ratio of the 424 Da isomers to the 326 Da isomers). The distribution of the other isomers was similar for the plastic samples, with increases in the relative amounts of the 376 and 400 Da isomers. These results warrant further study of large PAHs for their ability to discriminate between sources of PAHs.

**Figure 4:**
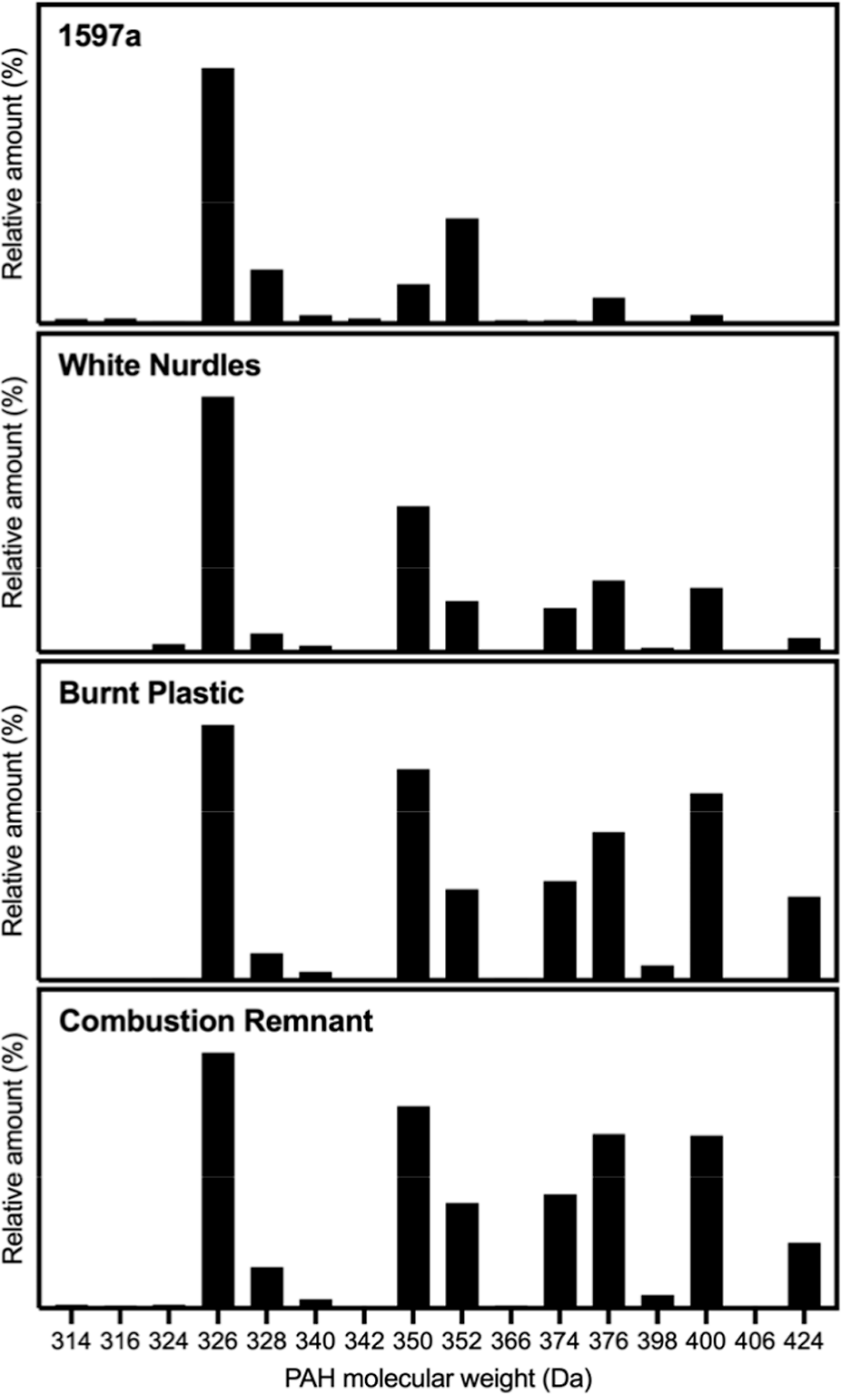
Comparison of the distributions of large PAH isomeric classes between SRM 1597a and the plastic samples. Values are the normalized integrated peak areas for the large PAH isomeric classes.

Quantitative analysis for the contents of 1,3,5-TPB in the white, burnt, and combustion remnant samples were 1.4, 0.2, and 0.2 ppb, respectively. The contents of 1,3,5-TPB in the pyroplastic samples (burnt and combustion remnant) were approximately an order of magnitude lower than in the white unburnt nurdles. This finding supports the need for multiple markers of plastic burning and pyroplastics, as well as further study of the formation conditions for 1,3,5-TPB during plastic burning.

## Discussion

The presence of large PAHs in environmental samples has been known for decades; however, research on them has, in part, been stymied by a lack of pure standards and by their inaccessibility via conventional approaches. We were able to detect large PAHs by using GC-APCI and relying on SRMs in lieu of standards for them. The measurement of large PAHs is now manageable.

The marked increase in large PAHs in pyroplastic field samples compared to unburned nurdles underscores the importance of these compounds in characterizing environmental pyroplastics. This significant difference in PAH content highlights the potential of large PAHs as practical markers for identifying and studying pyroplastics. In pyroplastics, these compounds are relatively abundant and positioned to provide diagnostic value. The fact that there are pure standards for only select compounds does not preclude the utility of large PAHs. As is the widespread practice in oil spill forensics, the interpretation of extracted ion chromatograms is valid and valuable. By focusing on high-molecular weight PAH compounds, researchers can gain a more comprehensive understanding of the chemical composition of pyroplastics, which is crucial for assessing their environmental impact and developing effective monitoring strategies. The detection of many large PAHs in both pyroplastic samples provides a promising list of candidate markers for polyethylene combustion. Given that polyethylene is one of the most widely produced and environmentally prevalent polymers, these markers can complement other chemical indicators (e.g., 1,3,5-TPB, 1,2,4-TPB, and tris­(2,4-di-*tert*-butylphenyl)­phosphate^41^) for detecting partially combusted plastics in environmental samples.

Our results support that 1,3,5-TPB can be specific to burning plastic, as it was present in the pyroplastic samples and was not detected in the coal tar SRM (a petrogenic source). Of note, 1,3,5-TPB was originally identified in the smoke produced when burning polyethylene bags and later in atmospheric particulate matter collected over burning sites,^40–42,56^ not within the remaining plastic residues (i.e., pyroplastic). We detected 1,3,5-TPB within the visibly unburnt plastic (white nurdles) and at greater amounts than in the partially combusted plastics (pyroplastics). This finding contrasts with the relative amounts of small and large PAHs detected in these materials,^39^ specifically the 424 Da isomers, which are present in greater relative amounts in the burnt plastic and combustion remnants. This outcome motivates further evaluation of 1,3,5-TPB and the 424 Da PAH isomers as the de facto markers of burning plastic, necessitating an investigation into the formation of 1,3,5-TPB during the heating and burning of plastic.

Factors such as temperature, oxygen availability, and the presence of other materials during combustion can influence the formation of specific PAHs.^1,8^ Additionally, environmental factors like exposure to sunlight, water, and microbial activity are expected to alter the chemical composition of pyroplastics over time.^39^ Therefore, differences in large PAHs observed between pyroplastic samples may be attributed to varying conditions during their transport and residence in the environment, as well as differences in the components and conditions of combustion. Understanding these variables is essential for interpreting the presence and concentration of large PAHs in environmental matrices and for developing accurate methods for their detection and analysis.

The relative increase of certain large PAHs in pyroplastic samples establishes the foundation for using these compounds as markers for environmental pyroplastics. Identifying common large PAHs provides a starting point for developing reliable markers of polyethylene combustion. However, the variability in large PAHs due to environmental and combustion conditions underscores the need for thorough validation and a deeper understanding of these markers. By investigating these interrelated points, researchers can enhance the detection and characterization of pyroplastics, thereby improving environmental monitoring and pollution management.

## Conclusions

GC-APCI-MS/MS proved a useful technique for detecting large PAHs in diverse environmental matrices, including extracts from pyroplastic field samples. Despite a lack of widely available analytical standards for large PAHs, NIST SRMs provided ample material for method development for their detection. Nonetheless, with the increasing research on microplastics in the environment and their relevance to human health, an environmental SRM with certified values for large PAHs is needed, and pyroplastics may provide such a source of material. Large PAHs and 1,3,5-TPB were detected in pyroplastic field samples and hold promise as chemical markers for pyroplastics, complementing other means for their detection in environmental samples (e.g., appearance and physical properties). Due to the accessibility of GC-APCI, the detection of large PAHs can be integrated into workflows for evaluating environmental samples, including those for microplastics.

## Supplementary Material



## Notes

Additional instrument and method details; MRM transitions for 16 large PAHs (Table S1); MRM transitions for 1,3,5-TPB (Table S2); calibration curve for 1,3,5-TPB (Figure S1); comparison of the resolved isomers for 302 Da in the SRMs and plastic samples (Figure S2); comparison of the resolved isomers for 374, 350, and 326 Da in the SRMs (Figure S3); comparison of the resolved isomers for 350, 374, and 398 Da in the plastic samples (Figure S4) (PDF)
