# Supplementary material for: Gas Chromatography-Atmospheric Pressure Chemical Ionization (GC-APCI) Expands the Analytical Window for Detection of Large PAHs (≥24 Ringed-Carbons) in Pyroplastics and Other Environmental Matrices
Source: ACS Omega. 2026 Feb 8;11(7):12321–9. doi: 10.1021/acsomega.5c11703 (PMC12947169; doi:10.1021/acsomega.5c11703)
Supplement: Supplementary file 1 [file ao5c11703_si_001.pdf]

Supporting Information

Gas chromatography-atmospheric pressure chemical ionization (GC-APCI) expands the analytical window for detection of large PAHs (≥ 24 ringed-carbons) in pyroplastics and other environmental matrices

Cara Megill[1], Douglas M. Stevens[2*], Christopher M. Reddy[3], Bryan D. James[1*], Robert K. Nelson[3], Frank L. Dorman[2,4]

[1]Department of Chemical Engineering, Northeastern University, Boston, MA 02115, USA
[2]Waters Corporation, 34 Maple Street, Milford, MA 01757, USA.
[3]Department of Marine Chemistry and Geochemistry, Woods Hole Oceanographic Institution, Woods Hole, MA 02543, USA.
[4]Department of Chemistry, Dartmouth College, Hanover, New Hampshire 03755, USA.

*Co-corresponding authors. Emails: doug_stevens@waters.com; b.james@northeastern.edu

Additional instrument and method details

The following source settings, gas flows and detector settings were used for the GC-APCI system:

Corona Pin (uA)	2.0
Source Temperature (°C)	150
Cone Gas Flow (L/Hr)	270
Auxillary Gas Flow (L/Hr)	200
Makeup Gas Flow (ml/Min)	350
Collision Gas Flow (mL/Min)	0.40
Detector Gain	0.20

Soft Transmission Mode: Disabled

Table S1. MRM transitions used for 16 large PAHs.

MRM Transition	Dwell (s)	Cone (V)	Collision Energy (eV)
1 : 314.10 > 310.10	0.002	50	60
2 : 314.10 > 312.11	0.002	50	70
1 : 316.10 > 312.10	0.002	50	90
2 : 316.10 > 314.10	0.002	50	70
1 : 324.10 > 320.10	0.003	60	90
2 : 324.10 > 322.10	0.003	60	70
1 : 326.10 > 274.05	0.003	60	100
2 : 326.10 > 322.10	0.003	60	90
3 : 326.10 > 324.10	0.003	60	70
1 : 328.10 > 324.10	0.003	60	90
2 : 328.10 > 326.10	0.003	60	70
1 : 340.10 > 336.10	0.003	60	90
2 : 340.10 > 338.10	0.003	60	70
1 : 342.10 > 338.10	0.003	60	90
2 : 342.10 > 340.10	0.003	60	70
1 : 350.10 > 346.10	0.004	60	90
2 : 350.10 > 348.10	0.004	60	70
1 : 352.10 > 348.10	0.004	60	95
2 : 352.10 > 350.10	0.004	60	75
1 : 366.10 > 362.10	0.004	60	95
2 : 366.10 > 364.10	0.004	60	75
1 : 374.10 > 370.10	0.005	60	100
2 : 374.10 > 372.11	0.005	60	80
1 : 376.10 > 372.10	0.005	60	95
2 : 376.10 > 374.10	0.005	60	75
1 : 398.10 > 394.10	0.005	60	95
2 : 398.10 > 396.10	0.005	60	75
1 : 400.10 > 396.10	0.005	60	95
2 : 400.10 > 398.10	0.005	50	75
1 : 406.10 > 402.10	0.005	60	95
2 : 406.10 > 404.10	0.005	60	75
1 : 424.10 > 420.10	0.006	60	100
2 : 424.10 > 422.10	0.006	60	70

Note that the collision energies are based on the use of N_2 collision gas.

Table S2. MRM transitions used for 1,3,5-TPB analysis.

MRM Transition	Dwell (s)	Cone (V)	Collision Energy (eV)
1 : 306.10 > 202.10	0.002	50	70
2 : 306.10 > 226.10	0.002	50	70
3 : 306.10 > 228.10	0.002	50	55
4 : 306.10 > 289.10	0.002	50	60
5 : 306.10 > 302.10	0.002	50	60
6 : 306.10 > 304.10	0.002	50	55

Note that the collision energies are based on the use of N_2 collision gas.

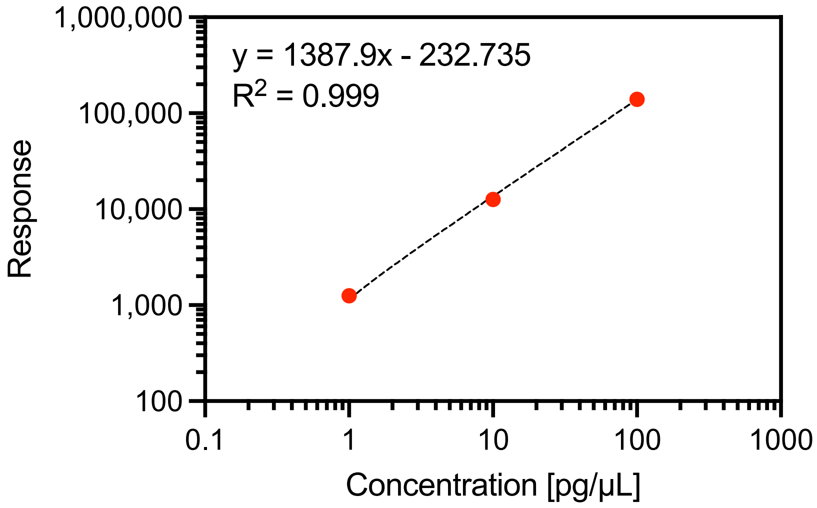

Figure S1. Calibration curve for the 1,3,5-TPB standard used to quantify that analyte in the NIST SRM 1597a and environmental pyroplastics.

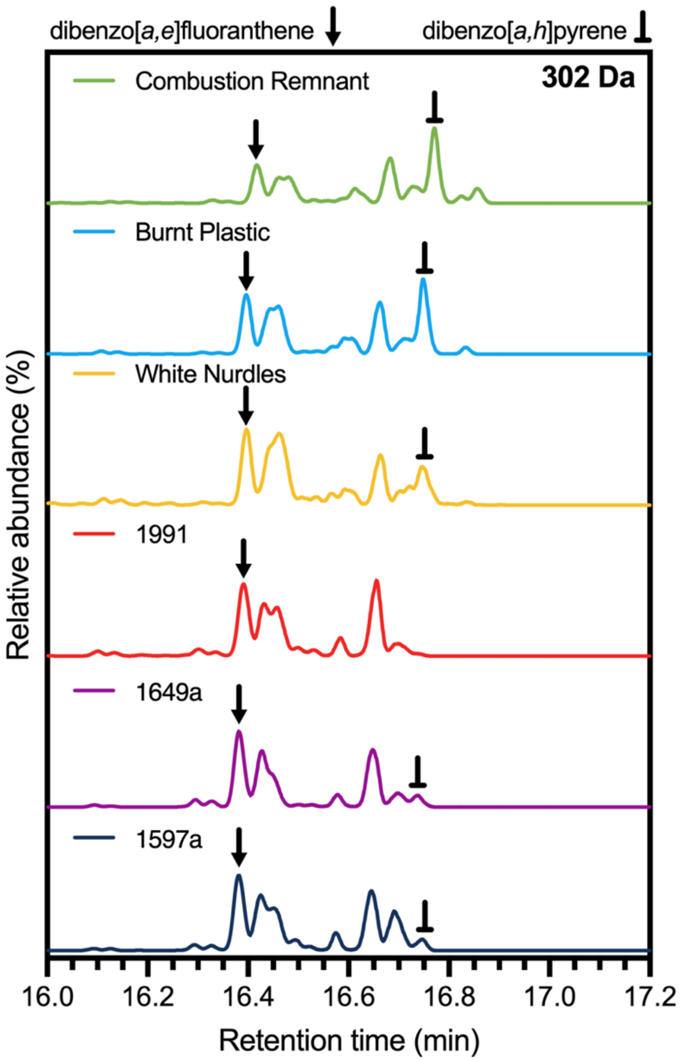

Figure S2. Comparison of the 302 Da isomers of SRMs 1597a, 1649a, and 1991, along with the white nurdles, burnt plastic, and combustion remnant samples collected from Sri Lankan beaches following the 2021 M/V *X-Press Pearl* ship fire and plastic spill.

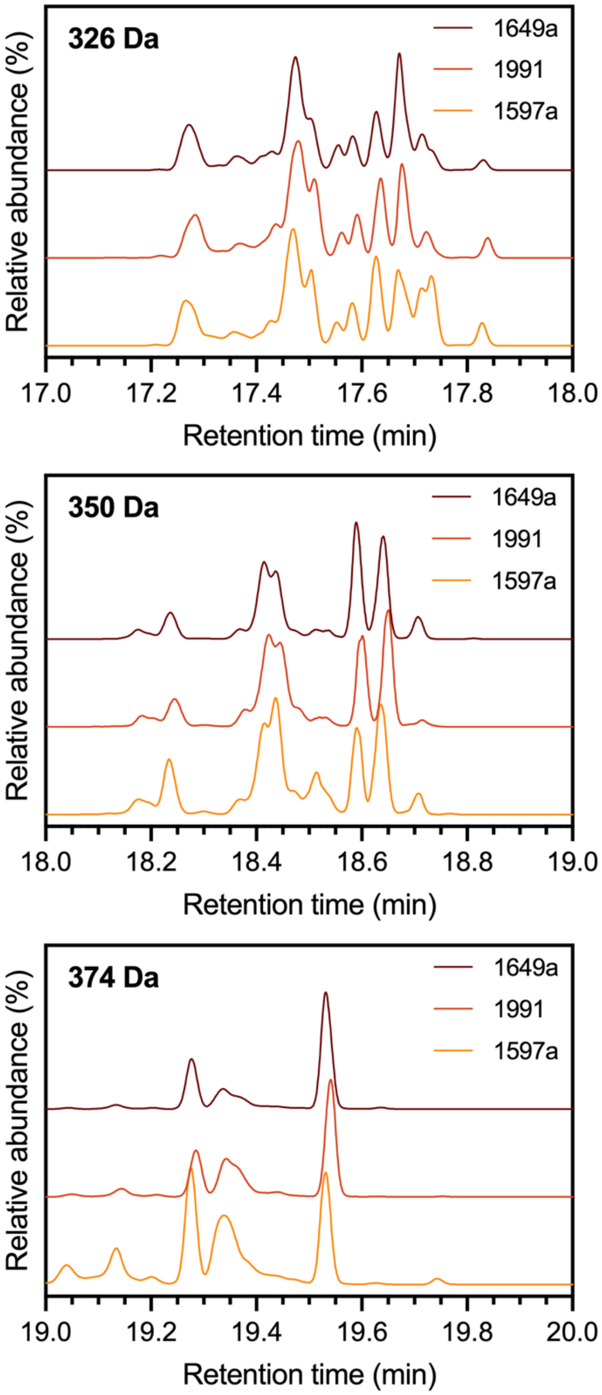

Figure S3. Comparison of the resolved isomers for 374, 350, and 326 Da of the three SRMs 1597a (coal tar), 1991 (mixed coal tar/petroleum), and 1649a (urban dust).

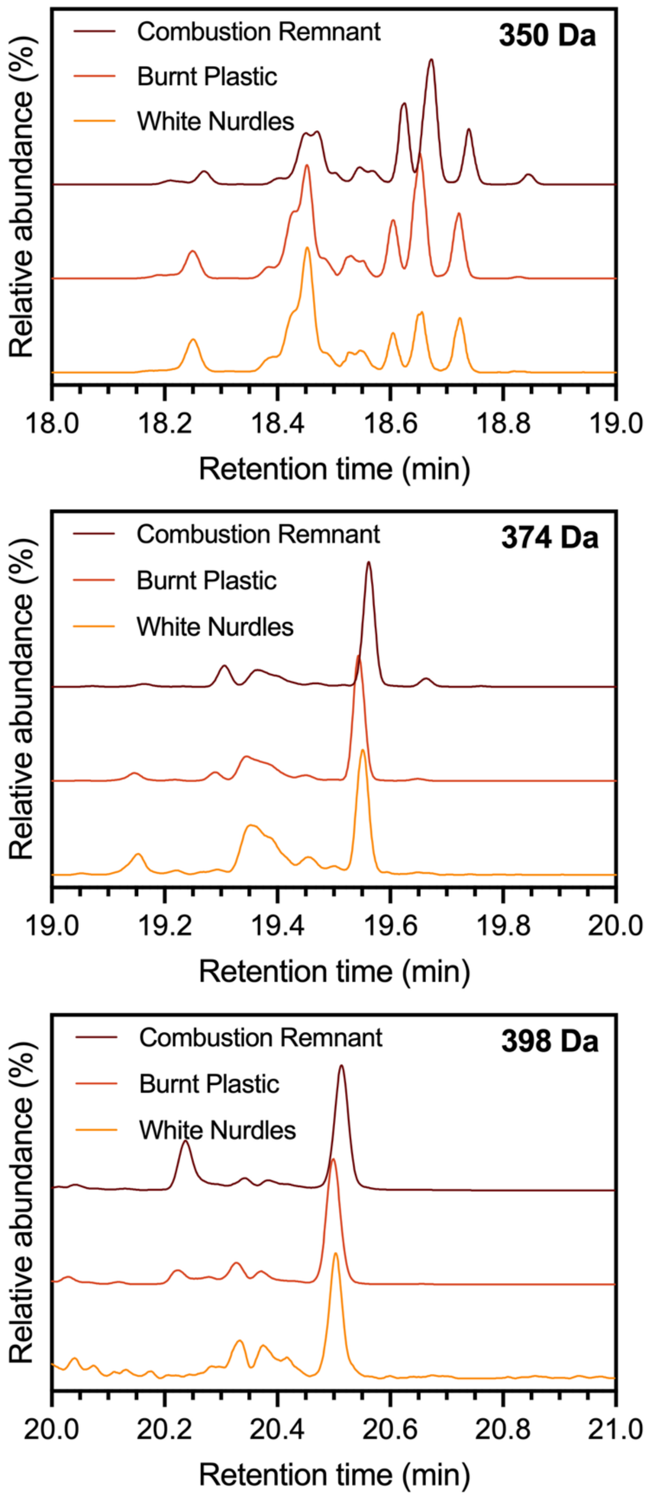

Figure S4. Comparison of the resolved isomers for 350, 374, and 398 Da of the white nurdles, burnt plastic, and combustion remnant pieces collected from Sri Lankan beaches following the 2021 M/V *X-Press Pearl* ship fire and plastic spill.
